# Predicting valuable missense variants with AlphaMissense in a multiple pulmonary infection patient

**DOI:** 10.1002/ccr3.8453

**Published:** 2024-01-30

**Authors:** Tianyuan Wang, Jindi Ma, Yuan Shu, Bao Hong, Zhouhan Wang, Yingfeng Lu, Xiaopeng Yu, Xi Huang, Yimin Zhang

**Affiliations:** ^1^ State Key Laboratory for Diagnosis and Treatment of Infectious Diseases, National Clinical Research Center for Infectious Diseases, Collaborative Innovation Center for Diagnosis and Treatment of Infectious Diseases, The First Affiliated Hospital, College of Medicine Zhejiang University Hangzhou China; ^2^ Department of Infectious Diseases Haining Pepole's Hospital Haining China; ^3^ Department of electrocardiogram, The First Affiliated Hospital, College of Medicine Zhejiang University Hangzhou China

**Keywords:** exome sequencing, machine learning, multiple infections, Phenolyzer

## Abstract

AlphaMissense is proficient in predicting the clinical classification of missense variants. we utilized AlphaMissense to find disease‐relevant variants within a polymicrobial pulmonary infection case. Exome sequencing was performed in this patient, and AlphaMissense and Phenolyzer were combined to investigate disease‐relevant variants screening from exome sequencing results.

## Graphical abstract

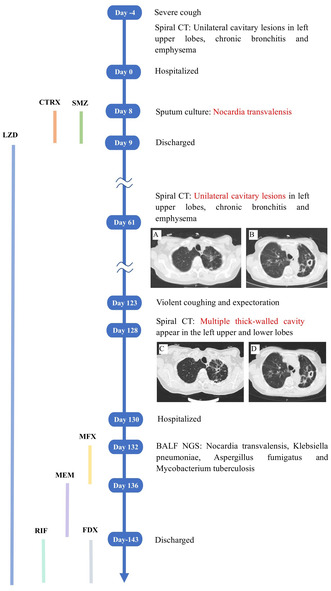

## 1 INTRODUCTION

The increasing quantity of gene–disease connections and the overlap in clinical characteristics among various genetic disorders create challenges in distinguishing between them. These factors can lead to a time‐intensive and laborious process when searching for a potential genetic diagnosis. For these disorders, a molecular‐guided diagnostic approach with genotype‐first emphasis followed by retrospective phenotyping may represent a more suitable strategy.^1^ With the increasing availability of next‐generation sequencing (NGS) and the more routine implementation of genomic sequencing in clinical practice, there is a heightened necessity to explore their effectiveness. Next‐generation DNA sequencing technologies such as whole‐exome sequencing (WES) have revolutionized the field of genomics. It allows an increasingly cost‐effective and high‐throughput method for complex and rare disease diagnoses.^2^, ^3^ These approaches prove helpful in diagnosing patients with atypical or insufficient clinical presentations.^4^ Despite NGS‐based methods have achieved significant success in mutation identification, practical challenges remain regarding the analytical accuracy of NGS approaches, especially in clinical contexts. Machine learning methods have the potential to bridge the gap in variant interpretation. AlphaMissense is an innovative technology designed to predict the pathogenicity of missense variants by integrating knowledge gained from both protein structure and evolutionary characteristics to achieve a comprehensive understanding. The predictions offered by AlphaMissense provide valuable insights on how variants impact protein function, aiding in identifying pathogenic missense mutations and previously undiscovered disease‐causing genes.^5^ An additional tool, Phenolyzer, can effectively prioritize disease‐associated variants identified in WES. Phenolyzer leverages pre‐existing biological knowledge and phenotype data to implicate genes associated with diseases.^6^

Cystic fibrosis (CF) is an autosomal recessive disease resulting from mutations in the CF transmembrane conductance regulator (CFTR) gene.^7^ In CF patients, it has been observed that monocytes and monocyte‐derived macrophages exhibit impaired phagocytic and antimicrobial functions when challenged by lung pathogens.^8^, ^9^, ^10^ As a result, the CFTR defect is categorized as a primary immunodeficiency disorder (PID) by the International Union of Immunological Societies.^11^

The plasminogen system is tightly controlled and safeguarded against dysfunction by activators and inhibitors. Plasminogen (PLG) serves essential physiological functions in fibrinolysis, cell signaling, and inflammatory regulation.^12^ Functional mutations in *PLG* may result in a phenotype associated with PLG deficiency. This deficiency is characterized by increased numbers of neutrophils and macrophages, reduced efferocytosis, and elevated IL‐6, which are associated with organ damage.^13^

Herein, we report a comprehensive analysis that encompasses the application of genomics filtering to WES data, as well as the predictions of disease‐associated genes using AlphaMissense and Phenolyzer to prioritize candidate genes based on phenotypic assessment. We employed Phenolyzer to analyze missense variants associated with the patient's phenotype. The detected missense variants were further prioritized based on their correlation, incorporating the use of AlphaMissense to predict the pathogenicity of these variants. Ultimately, within the subset of missense variants exhibiting high correlation and a high likelihood of pathogenicity, candidate genes were selected based on their biological features. This approach provides a comprehensive strategy for identifying the most probable disease‐causing gene in the context of the patient's phenotype.

## 2 MATERIALS AND METHODS

### 2.1 Clinical ethics and data

Samples were obtained from Haining People's Hospital. Clinical data were derived from the medical record system of Haining People's Hospital. Informed consent was obtained from the patient. This case has been approved by the Ethical Review Committee of Haining People's Hospital.

### 2.2 Exome sequencing and analysis

Genomic DNA extracted from peripheral blood for each sample was fragmented to an average size of 180–280 bp and subjected to DNA library creation using established Illumina paired‐end protocols. The Agilent SureSelect Human All ExonV6 Kit (Agilent Technologies) was used for exome capture according to the manufacturer's instructions. The Illumina Novaseq 6000 platform (Illumina Inc) was utilized for genomic DNA sequencing in Novogene Bioinformatics Technology Co., Ltd to generate 150‐bp paired‐end reads with a minimum coverage of 10× for 99% of the genome (mean coverage of 100×). We filtered the variants that did not reside within highly conserved regions or had a frequency exceeding 1% in any of the four frequency databases: Thousand Genomes Project (1000g_all), ESP6500 database (esp6500si_all), gnomAD data (gnomAD_ALL and gnomAD_EAS). And we retained variants that were predicted to be deleterious or to have an impact on splicing (Table S1).

### 2.3 AlphaMissense predicting variant pathogenicity

The single nucleotide polymorphisms (SNPs) identified from WES were compared with the AlphaMissense model. AlphaMissense utilizes databases containing relevant protein sequences and considers the structural context of genetic variants to assign a score ranging from 0 to 1, indicating the probability of a variant being pathogenic. Specific threshold score values are established to classify a variant as either “likely pathogenic,” “ambiguous,” or “likely benign.”

### 2.4 Phenolyzer evaluating gene prioritization

Variants classified as “likely pathogenic” and “ambiguous” by AlphaMissense were analyzed by Phenolyzer. HPO terms were manually inputted into the Phenolyzer web interface (https://phenolyzer.wglab.org) for evaluation. The output file includes a generated gene‐disease‐term interaction network and a bar plot featuring highly ranked genes with their corresponding normalized scores, available for download.

## 3 RESULTS

### 3.1 Case presentation

Here, we report a case of a 70‐year‐old female patient who has had chronic cough and sputum production for 10 years. At 66 years of age, she was diagnosed with bronchiectasis. As past history, she had hypertension diagnosed 5 years earlier. She claimed no alpha‐1‐antitrypsin genotype, nor a smoking history, history of atopy, asthma, rheumatic diseases, family history of immunodeficiency, cystic fibrosis, or plasminogen deficiency.

She presented in April 2021 with a 1‐week history of cough and sputum onset (day‐4). She did not receive any antibiotic treatment before being admitted to the hospital. On admission, she was ill‐appearing, with an oxygen saturation of 96% on room air. Her chest inspection revealed a barrel chest, and chest auscultation revealed moist rales over the inferior zone of the left lung. Laboratory examinations revealed a C‐reactive protein level of 21.4 mg/L. Anti‐U1RNP and anti‐SM antibodies displayed weak positivity, with normal globulin levels, thyroid function, and no observed deficiency in T cells, B cells, or complement. Computed tomography (CT) scan revealed a cavitary lesion in the upper lobe of the left lung, chronic bronchitis, and emphysema (day 0). The spleen ultrasound did not indicate any abnormalities. No HIV infection was detected.

Given that a 5‐day course of sulfamethoxazole (800 mg/three times daily) and ceftriaxone (2000 mg/day) treatment for sputum culture showed growth of N. transvalensis had improved her symptoms (day 8), she was discharged 3 weeks after treatment. In an outpatient setting (day 13), linezolid (300 mg per day) was added.

On day 61, the chest radiograph showed multiple thick‐walled cavities in the upper and lower lobes of the left lung and bronchiectasis with infection in both lungs, despite the administration of linezolid for the progression of pulmonary infections.

Only 4 months after the administration, her symptoms worsened again. She was admitted to our hospital with complaints of severe cough and sputum and was febrile to 38°C (day 130).

A detailed medical investigation was scheduled. Laboratory examinations revealed an increased white blood cell count and neutrophil cell count of 11.8 × 10^9/L and 9.43 × 10^9/L, respectively. C‐reactive protein level and procalcitonin were 241 mg/L and 0.28 ng/mL, respectively. CT scan indicated that multiple thick‐walled cavities in the upper and lower lobes of the left lung field worsened again, and new consolidation occurred in the left upper lung field.

Despite adding moxifloxacin (400 mg/day) (day 132) and re‐administered linezolid, her cough and sputum worsened, and her body temperature persisted over 38°C. So, she was admitted for further examinations.

**FIGURE 1:**
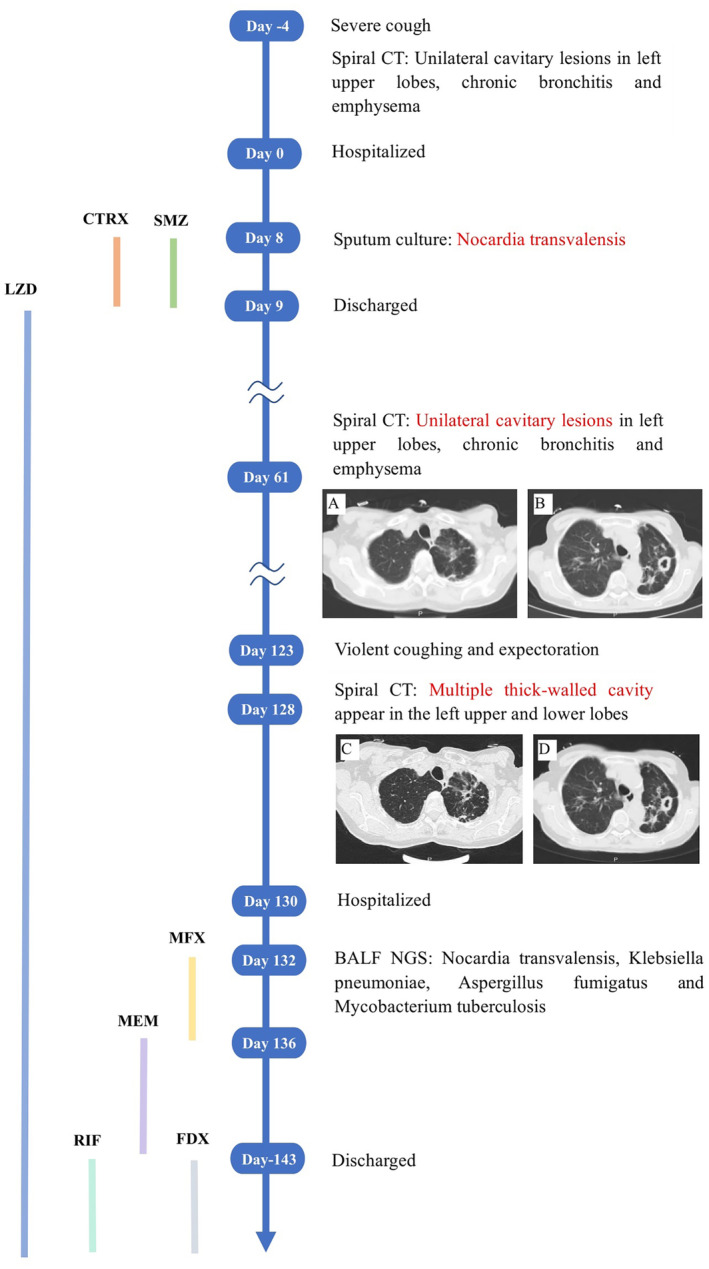
Clinical course of the patient. Sulfamethoxazole and ceftriaxone (day 12–17), linezolid (day 18), moxifloxacin (day 136–140), meropenem (day 141–161), faropenem and rifampicin (day 161). (A, B) Contrast‐enhanced CT of the pulmonary at day 65. (C, D) Contrast‐enhanced CT of the pulmonary at day 132. BALF, bronchoalveolar lavage fluid; CT, computed tomography; CTRX, ceftriaxone; FDX, faropenem; LZD, linezolid; MEM, meropenem; MFX moxifloxacin; NGS, next‐generation sequencing; RIF, rifampicin; SMZ, Sulfamethoxazole.

A complex of Nocardia transvalensis, Klebsiella pneumoniae, Aspergillus fumigatus, and Mycobacterium tuberculosis was detected from the next‐generation sequencing of bronchoalveolar lavage fluid. Culture of bronchoalveolar lavage fluid showed growth of Nocardia transvalensis and Klebsiella pneumoniae. Sputum culture showed growth of Candida albicans and Stenotrophomonas maltophilia was observed. Intravenous administrations of meropenem (500 mg, three times daily) and oral administrations of linezolid (300 mg per day) (day 136) were started based on the result of NGS. Her symptoms improved gradually. The course of intravenous meropenem treatment was completed, and she was discharged 3 weeks after starting the treatment. Faropenem, rifampicin (450 mg per day), and linezolid (200 mg, three times daily) were continued in an outpatient setting. Moreover, she was suggested to follow up 1 month after the initial treatment (Figure 1).

### 3.2 WES filtered variants in the exon region

As a result, 336 SNPs were screened (Table S2). Regarding amino acid translation, there were 297 missense, 10 stop‐gain SNPs, one frameshift insertion, five frameshift deletions, five non‐frameshift insertions, six non‐frameshift deletions and one stop‐gain for indel variants.

### 3.3 Predicting pathogenic variants with AlphaMissense

From the findings in the WES genes, 48 missense variants are predicted as likely pathogenic, 201 variants as likely benign, and 31 variants as ambiguous. Variants classified as “likely pathogenic,” and “ambiguous” are selected as candidate genes for the subsequent analysis step.

### 3.4 Phenolyzer discovered interaction between variants and phenotypes

**FIGURE 2:**
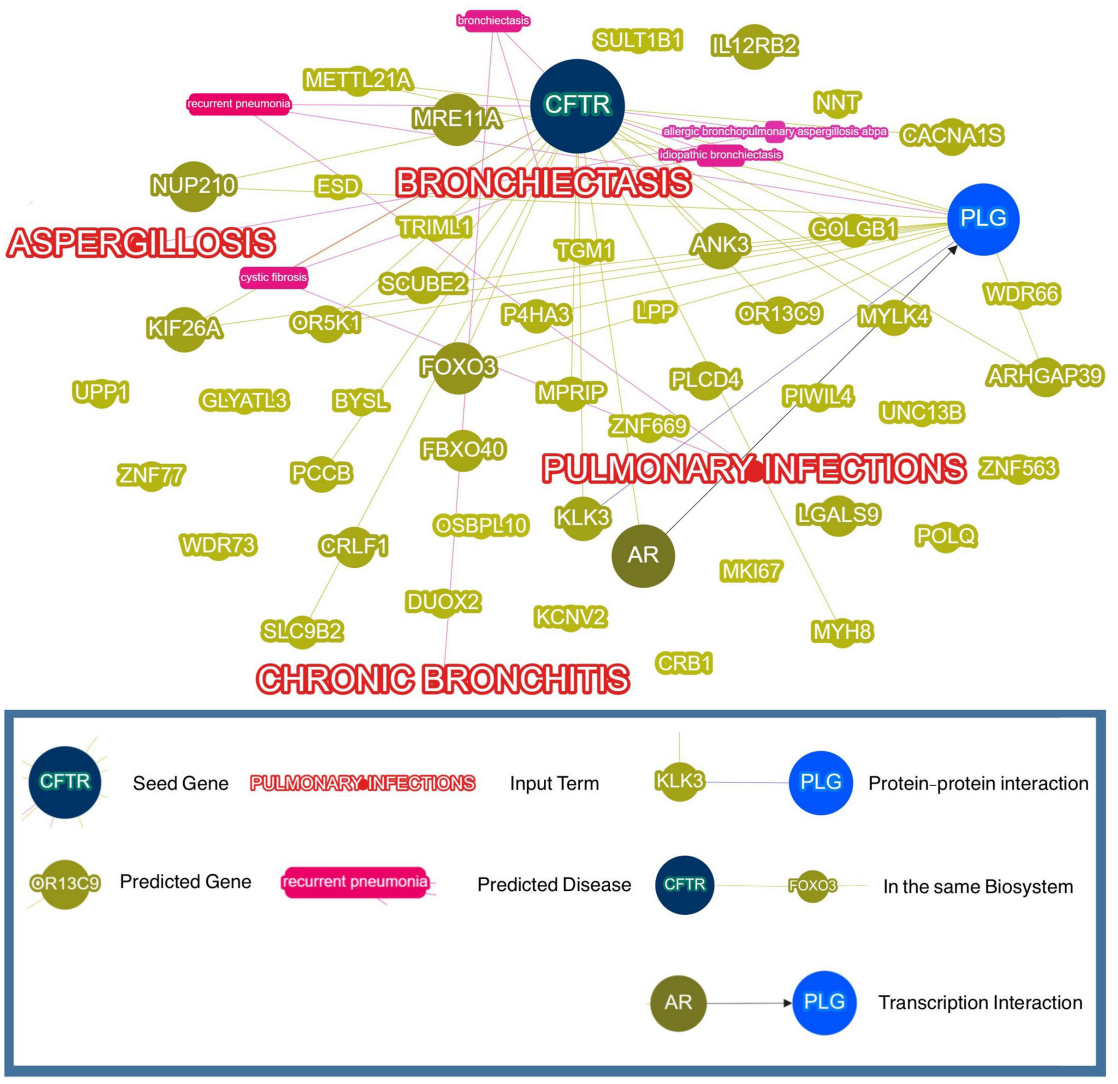
Phenolyzer network analysis of gene discoveries, HPO terms, and disease categories. The most disease‐relevant genes are displayed as seed genes. Yellow edges denote that the two node genes are in the same biosystem, blue lines denote interactions between proteins encoded by two genes, and black edges with arrows denote transcriptional interactions between two genes, with the arrow pointing from the transcription factor to the target.

“Multiple pulmonary infections,” “klebsiella infections,” and “tuberculosis “were entered as phenotype into the Phenolyzer web interface for analysis. The Phenolyzer network analysis is depicted in Figure 2. The most disease‐relevant genes are presented as seed genes. Among all genes detected by WES, *CFTR* and *PLG* are the most reliable genes, playing a critical role in pulmonary infections and recurrent pneumonia. The Phenolyzer score for each gene is normalized to a range from 0 to 1, where a higher score indicates a greater influence on the diagnosis of the associated disease. “*CFTR*” possesses a Phenolyzer score of 0.929, while “*PLG*” scores 0.47. The *CFTR* variant was detected at position 117182149 on chromosome 7: [NM_000492] c. C1196G. And the *PLG* variant was found at position 161134046 on chromosome 6: [NM_000301] c. c.G436A.

## 4 DISCUSSION

With the development of gene sequencing technology, more than 4 million missense variants have been detected. However, only about 2% have been classified as pathogenic or benign variants clinically.^5^ Classifying the remaining vast number of variants is a significant challenge that waits to be addressed. With the great achievement of deep learning AI model, AlphaFold2, in predicting protein structures, AlphaMissense was successfully developed to classify 32% as likely pathogenic and 57% as likely benign using a cutoff that yields 90% precision. This represents great progress in classifying the significance of observed missense variants.

In the present case report, the patient was otherwise healthy and presented with multiple infections caused by Nocardia transvalensis, Klebsiella pneumoniae, Aspergillus fumigatus, and Mycobacterium tuberculosis. With WES, we filtered 336 SNPs and 43 indels in this particular case. However, WES may not always identify the precise disease‐relevant variations.

The impact of the majority of these genetic variants remains elusive, which restricts their clinical applicability and actionable insights. Fortunately, machine learning approaches that such as AlphaMissense can predict disease‐causing mutations from identified genetic alterations, represent a promising first stride toward harnessing the potential of personalized genomic medicine. HPO has established itself as a standardized method for phenotype comparison. The creation of phenotype analysis tools, exemplified by Phenolyzer, facilitates the connection between phenotypes and genetic variants present in numerous databases. When selecting specific patient characteristics, it enables users to access a wealth of clinical and scientific information related to the conditions associated with the selected features. Individually, neither method is optimal for clinical diagnosis. However, they synergize with each other, providing valuable support to clinicians in the prioritization of variants for rare disease diagnostics.

The most valuable candidate gene identified in our study, *CFTR*, is recognized as the cause of CF, an autosomal‐recessive disorder that significantly impacts life span. Patients diagnosed with CF are susceptible to infections from a wide range of pathogens. In an ecological study investigating the impact of individuals carrying single pathogenic variants of CF, that is, CF carriers, on the spread and fatality of COVID‐19 across 37 countries, it was revealed that the prevalence of CF significantly and directly correlated with both the prevalence of COVID‐19 and its case fatality rate.^14^ Moreover, Polgreen et al. identified that CF carriers exhibit increased susceptibility to respiratory infections and are more inclined to use antibiotics for treating respiratory infections compared to the general population.^15^ Expanding on this, a more extensive study, involving 19,802 CF carriers and 79,208 controls, demonstrated that the incidence rates of various respiratory infections, including nontuberculous mycobacterial, aspergillosis‐associated, and Pseudomonas infections, were higher among CF carriers than in the control group.^16^ The *CFTR* carrier status has the potential to exacerbate lung disease in PID patients who already suffer from lung ailments, which has been observed in CF. The heterozygous carriage status of *CFTR* might act as a gene modifier in PID.^17^ Furthermore, the CFTR defect is classified as a PID based on the dysfunction of monocytes and monocyte‐derived macrophages. While the most severe forms of PIDs typically manifest in early childhood, a significant proportion of patients present in adulthood. Among PID patients over the age of 30, approximately 80% present with infection as the initial manifestation, without evident immune dysregulation.^18^ These adult‐onset cases often lack a family history of PID and exhibit a diverse range of clinical phenotype.^19^ However, genetic diagnosis poses challenges in sporadic PID cases, and the significance of genetics remains unclear.

The candidate gene PLG, identified in our study, also performs crucial functions in bacterial infections. Pathogenic bacteria, including pneumococci and Streptococcus pyogenes, exhibit specific interactions with elements of the fibrinolytic pathways, allowing them to propagate within the host and evade the host's inflammatory immune response.^20^, ^21^ Staphylococcus aureus, for instance, has the capability to capture PLG from human plasma. This allows it to generate plasmin, which in turn cleaves tissue components, thereby promoting the spread of bacteria in infected tissues.^22^ Furthermore, in patients with septic shock, PLG levels were reduced compared to those in patients with sepsis. This observation demonstrates a negative correlation between the severity of sepsis and PLG levels.^13^ It confirms the protective effect of PLG against sepsis‐induced lethality and enhances the protective effects of antibiotics.

According to the relevance ranking provided by Phenolyzer, we examined the biological backgrounds of additional genes. The androgen receptor (AR), with a score of 0.33, belongs to the steroid subfamily of nuclear receptors and plays a crucial role in male development and tissue maintenance. Mutations in AR are associated with the conditions such as prostate cancer.^23^ The DNA repair nuclease MRE11A, scoring 0.19, is responsible for maintaining metabolic competence by safeguarding mitochondrial DNA. In cases of rheumatoid arthritis, MRE11A deficiency in T cells disrupted mitochondrial oxygen consumption, leading to suppressed ATP generation.^24^ Despite their intriguing functions, these genes exhibit relatively low correlation with the patient's phenotype.

This methodology for screening variants and identifying candidate genes, described in our study, relies upon the expertise and judgment of clinical practitioners. It is necessary for clinicians to accurately input the patient's phenotypes into Phenolyzer; otherwise, the genes output may not conclusively represent the pathogenic genes for the patient. Additionally, despite the substantial reduction in the array of candidate genes facilitated by this methodology, clinicians must integrate their experiential insights to pinpoint the most probable pathogenic gene.

## 5 CONCLUSIONS

This case demonstrates that using a Phenolyzer approach offers the benefits of cost reduction and a simplified workflow. Additionally, the combination of AlphaMissense enhances the efficiency in identifying of valuable missense variants in patients. The accurate characterization of these variants allows healthcare professionals to customize treatment strategies based on the specific genetic profile of each patient, thereby maximizing therapeutic efficacy while minimizing potential adverse effects. Additionally, there is a need for a comprehensive validation study on a larger scale, encompassing diverse patient populations. This study would be essential to evaluate the generalizability and robustness of the Phenolyzer and AlphaMissense combination across various genetic backgrounds and clinical scenarios. This would contribute to establishing the reliability and broad applicability of the proposed approach.

## AUTHOR CONTRIBUTIONS

**Tianyuan Wang:** Software; writing – original draft. **Jindi Ma:** Data curation; writing – original draft. **Yuan Shu:** Resources. **Bao Hong:** Resources. **Zhouhan Wang:** Investigation; visualization. **Yingfeng Lu:** Funding acquisition; supervision. **Xiaopeng Yu:** Project administration; supervision. **Xi Huang:** Investigation. **Yimin Zhang:** Conceptualization; methodology; writing – review and editing.

## FUNDING INFORMATION

This research was funded by the National Key Research and Development Program of China, grant number 2021YFC2301900‐2021YFC2301901, Key Research and Development Program of Zhejiang, grant number 2022C03125, and the Fundamental Research Funds for the Central Universities, grant number 2022ZFJH003.

## CONFLICT OF INTEREST STATEMENT

The authors declare no conflict of interest.

## INSTITUTIONAL REVIEW BOARD STATEMENT

The study was approved by the Ethical Committee of Haining People's Hospital (protocol code 2021E75 and date of approval: 2 September 2021).

## CONSENT STATEMENT

Written informed consent was obtained from the patient to publish this report in accordance with the journal's patient consent policy.

## Supporting information


Table S1:

Table S2:

## Data Availability

The data that support the findings of this study are available from the corresponding author upon reasonable request.
